# The significance of information after discharge for colorectal cancer surgery–a qualitative study

**DOI:** 10.1186/s12912-015-0086-6

**Published:** 2015-06-05

**Authors:** Maria Lithner, Rosemarie Klefsgard, Jan Johansson, Edith Andersson

**Affiliations:** Department of Health Sciences, Faculty of Medicine, Lund University, Box 157, 221 00 Lund, Sweden; Skåne University Hospital, SUS Lund, 221 85 Lund, Sweden; Department of Surgery, Skåne University Hospital, SUS Lund, 221 85 Lund, Sweden

## Abstract

**Background:**

The aim was to explore patients’ experiences of information and their information needs after discharge for colorectal cancer surgery.

**Methods:**

Thirty one interviews were performed with sixteen patients during the first seven weeks at home after discharge. Patients were included from three hospitals in the south of Sweden, two of which used an enhanced recovery programme.

**Results:**

*Trying to regain control in life by using information* was the overall theme emerging from the interviews. Patients experienced the bodily changes after surgery and the emotional impact of the cancer disease, and these combined experiences seriou/sly affected their ability to manage their daily lives. They both needed, and were in search of, information to increase participation in their own cancer trajectory and to facilitate the regaining of some measure of control in their lives. Waiting for different kinds of information increased the anguish and fear in the face of an unknown future.

**Conclusions:**

This study showed that receiving information was vital when patients tried to regain control in life after colorectal cancer surgery. The information was necessary in order to facilitate and manage the transition from hospital to home, and the need varied between different transitions. Patients needed more information to manage the daily life at home, but also to understand what the cancer disease really meant to them. This suggests a need for patients to participate more actively in the information and the discharge planning.

## Background

When patients leave the hospital for home after cancer surgery they are in an intensive and vulnerable phase of their cancer trajectory [1]. For patients with colorectal cancer (CRC) the initial diagnosis phase focusing on prognosis and treatment decisions rapidly transfers to the treatment phase with surgery [2]. Patients are known to change their preferences for information across the cancer trajectory and express the greatest need for information in the treatment phase [3]. In an interview study performed 6 weeks after CRC surgery patients’ main concern was better access to information and specialist advice in the early days after discharge [4]. This combination calls for further studies into patients’ information needs in relation to discharge and the first period at home. In order to gain a more comprehensive understanding of patients’ experiences, an additional interview could be used so as to enhance the depth and variation in the results.

The existing tendency of shorter lengths of stay in hospital for patients with CRC can result in less time to assimilate information and to prepare for discharge [5, 6]. More than half of patients with CRC are only slightly or not at all recovered when they leave the hospital, and suffer from pain, nausea and fatigue that would affect the ability to absorb information [7]. Few studies have focused on this first period at home and increased knowledge of patients’ needs during this phase are important to improve surgical cancer care.

The importance of information is well known and patients’ right to information is widely acknowledged, but to actually provide information that corresponds to patients’ information needs is still a great challenge, since both the need for, and the recall of, information differs between individuals [8]. There are other factors also known to motivate patients’ information seeking when facing a cancer diagnosis [9]. Demographics, personal experience of cancer among family or friends and the importance and usability of information for the person at that point, gives rise to different needs. Despite this knowledge, the importance of studying patients with different cancers and their needs at various phases in their trajectory still remains important in order to improve the clinical outcome for each group.

Earlier research in this area reveals unsatisfied information needs among patients after discharge for CRC surgery [4], indicating that more knowledge is needed on what their information needs are, how they want health care professionals to present it and where in the early recovery period information is most deficient.

### Aim

The aim of this study was to explore patients’ experiences of information and their information needs after discharge for colorectal cancer surgery.

## Method

Thirty one interviews were performed with sixteen patients during the first seven weeks at home after discharge from hospital for colorectal cancer surgery. The focus of the interviews was on how patients experienced the information they had received and what their information needs were. This study was designed so as to comprise both the initial period at home and the period after the postoperative visit when the results from the tissue samples and further treatment had been discussed.

### The context of providing information

The information differed to some extent since two of the three hospitals used the Enhanced Recovery Programme (ERP) during the time of this study [10]. The ERP protocol aims to reduce the physiological and psychological stress of surgery in order to enable rapid recovery, decrease complications and shorten the length of stay in hospital [11–13]. In these hospitals the information was both verbal and written [10]. In the third hospital, the focus was on unstructured, verbal information.

#### Discharge information

The procedures for discharge information were similar in all three hospitals. The information was verbal and unstructured. Some patients met a surgeon in the patient’s room or in a separate room where they would provide discharge information. The information could include a repetition of what had been done during surgery and a prescription of pain medication. The RN and the physiotherapist talked to patients before their discharge but did not provide structured information.

#### Post-discharge information

The post-discharge visit at the surgical clinic was similar in all three hospitals and information was given verbally by a surgeon about one month after discharge. Some patients were then recommended further treatment with chemo-therapy and those patients received an appointment with an oncologist and an RN at the oncology clinic before the therapy started.

### Patients

Ten men and six women were interviewed. The age varied between 50 and 82 years (mean 66.6, SD 10.1). Their occupations covered both white-collar and blue-collar work. Of the 16 patients, 15 lived together with a spouse/partner and one with an adult child. Ten of the patients had co-morbidities like heart or lung disease, diabetes or orthopaedic disease and had earlier experiences of health care. Eight patients were offered treatment with chemo-therapy.

Adult patients who had surgery for colorectal cancer without receiving a stoma at three hospitals in southern Sweden were evaluated for inclusion in a larger prospective study, [14]. Out of 161 consecutive patients, 30 did not want to participate, most of them indicating that they were too tired. Thirty-one patients could not participate due to other reasons like benign tumours, complications and administrative problems. From the final 100 participants 16 were included in an additional part with interviews. The inclusion of these interview patients were purposeful in order to increase variation in age and care (ERP or not). They signed written informed consent before the interviews started.

At the time of the second interview one patient had received information that the diagnosis was a benign tumour and the inclusion criteria was not fulfilled, thus the second interview of that patient was excluded from the analysis.

### Interviews

All the 31 interviews were carried out between April 2007 and January 2009 by the first author (ML). They were transcribed verbatim and turned into 431 pages of text in all, varying between 2.5 and 33.0 pages per interview (A4 format, single-spaced). The interviews were performed individually in the person’s home and on two occasions; 1–2 weeks after discharge and a second time, 5–7 weeks after discharge. The interview questions were semi-structured and similar at both interviews. They started with a question such as: Can you tell me about the information you received before discharge, and what you do consider to be important information? An interview guide was used and embraced the following areas: Experiences related to receiving information before discharge. What information do you need now after surgery and discharge? What information is of the most importance? How you want to receive the information?

### Analysis

Qualitative content analysis with a conventional approach was used to analyse the text. The conventional approach is used when the study design is aimed to describe a phenomenon where the existing literature is limited [15]. Both manifest and latent analysis were used [16].

All text from the first interviews was read as a whole several times and during the discussions different areas emerged that turned into eight subcategories. Sentences or pieces of the text (meaning units) related to the aim of the study were identified in the text and labelled with codes. The meaning units, codes and subcategories were discussed several times and lead to three tentative categories. The whole text was read again to see if the categories corresponded to the original meaning of the text. The categories were analysed critically and questioned so as to arrive at a reasonable understanding.

After this phase was completed, the text from the second interviews was read and analysed in the same way as from the first. The categories and subcategories from the first interviews remained unchanged, but one subcategory concerning treatment with chemo-therapy was added. The meaning units related to the subcategories were extracted by using the computer programme NVIVO10 [17]. In the last step, the authors reflected on the findings and came to an agreement on an overall theme embracing how patients’ perceived their experiences of information and their need for information after discharge for colorectal cancer surgery.

### Ethical considerations

Every precaution was taken to protect the privacy of the participants and the confidentiality of their personal information and to minimize the impact of the study on their physical, mental and social integrity [18]. If the interview situation highlighted any need for further emotional support, a contact with a counsellor was offered. The participants could also relinquish the study at any time. This project was reviewed by the Regional Ethics Review Board, Lund, Sweden; Reg 558/2006.

## Results

An overall theme emerged from the interviews *Trying to regain control in life by using information* (Table 1). Patients experienced the bodily changes after the surgery, the emotional impact of the cancer disease and these combined experiences seriously affected their ability to manage their daily lives. They therefore needed, used, and were in search of, information to handle the physical and emotional challenges they experienced when coming home after colorectal cancer surgery. In the text, patients frequently refer to the period of waiting, before receiving different kinds of information and describe the anguish of fearing the unknown future and of not knowing what to expect next.

**Table 1:** Interpretation of patients’ experiences

Theme: trying to regain control in life by using information	
Categories	Subcategories
Using information to make daily life work	Managing symptoms and self-care at home
	Waiting for information about the next step
	Needing information to handle the chemo-therapy
Wanting to partake in the information	Expressing a desire for planning and participation
	Wishing to receive honest and straightforward information
	Obtaining information as part of a mutual meeting
Needing information to manage the worries and make the disease comprehensible	Understanding the cancer disease and what it means
	Expressing fear for the future
	Lacking information leads to anxiety and feelings of insecurity

### Using information to make daily life work

The following subcategories were brought together in this category; *Managing symptoms and self-care at home*, *Waiting for information about the next step* and *Needing information to handle the chemo-therapy*.

#### Managing symptoms and self-care at home

The patients expressed surprise over the lack of instructions when they left the hospital and wanted to know what they could do to enhance their recovery. They felt healthier when they could take an active part in the self-care and information was a prerequisite for that. They lacked information on how long time the recovery would take and when they could return to work and normal activity. Information on how to improve their physical fitness with training and exercise was described as important. Knowing more about what to eat and drink, how the bowel function and weight could be affected by the surgery and how to remove the suture and care for the wound were also considered as important information. Some patients were concerned about how to take medications and painkillers and some received incorrect prescriptions.

*If you don’t ask them yourself, things like what kind of food to eat, then you wouldn’t know a thing; rather it was more like a frisky, well, just carry on as usual thing, ok, well then why didn’t they say so, or write a note or something… (1 M)*

#### Waiting for information about the next step

The periods of waiting were times when the patients thought a lot about what the next step would mean to them. Before the post-discharge visit at the surgical clinic, patients had many thoughts concerning the analysis of the tissue sample and how these results would affect their future. Another period of waiting, the one between the visits at the surgical and oncological clinics, was experienced as a gap when they didn’t know where to turn with questions and concerns. They wanted the transfer to the oncology clinic to be faster and better coordinated. It was easier to handle the period of waiting if the time and day for the visit were known at an early stage.

#### Needing information to handle the chemo-therapy

The need for chemo-therapy was experienced as a new setback after already having tried to manage the cancer disease and the surgery. Patients needed time to adjust to this new information and they expressed fear, both of the side effects of the chemo-therapy and of the cancer disease itself, since the need for chemo-therapy made the gravity of the cancer more evident. The patients wanted to understand more about what chemo-therapy really is and how to receive it. They pondered over the possible side effects that might appear such as fatigue, nausea and losing hair. They also worried about the period of waiting before starting the therapy being too long, and how this might affect the development of the disease.

*What’ll my further treatment be like?…I mean I want treatment as soon as possible, and the right treatment…the big issue is after all the treatment I’ll be getting here from now on, that’s what it’s all about, and that it works, everything else fades away … (3 M)*

In contrast to this, patients who did not need further treatment with chemo-therapy were satisfied with the received information and had very few questions concerning the present and the future.

### Wanting to partake in the information

This category included the subcategories; *Expressing a desire for planning and participation, Wishing to receive honest and straightforward information* and *Obtaining information as part of a mutual meeting.*

#### Expressing a desire for planning and participation

A few patients were satisfied with the discharge information but most of them described the last day in the hospital as confusing and stressful. They did not know in advance what to expect that day, nor the time that they would actually be going home, who they could talk to or what the information would contain. They wanted to take an active part in the preparation but lacked information about the discharge procedures and therefore had a hard time participating in the process. The discharge was contrasted to the preoperative preparation, where the preoperative information were described as being calmer and easier to follow.

*The guy who was supposed to handle the discharge, he came at about half past three, I realize that they’ve got a lot to do, but it would’ve been good to know that sort of thing, you can take it easy now because it won’t be until late this afternoon…I would most of all have wanted a schedule, preferably the day before, but I suppose they have a shortage of beds and need to get rid of people, I think, I understand that too but if you’d had a schedule then you could’ve phoned home or to someone else the day before and said the times, so then they could’ve had a car standing there at such and such a time, that would be great (1 M)*

At the discharge, patients wanted to meet the surgeon who performed the operation and wanted the surgical procedure to be clarified with a drawing. They wanted the information to be both verbal and written, and to include the opportunity to ask questions.

To be able to influence both the content and quantity of the information was also important. Some patients wanted to receive most of the information early in the process and others wanted it to be divided into smaller parts. The importance of having a contact person both in the surgical and in the oncology clinic to get in touch with for further information was also emphasized.

#### Wishing to receive honest and straightforward information

To receive honest and straightforward information created a sense of confidence in the person who gave that kind of information. Other patients expressed a fear that healthcare professionals might withhold important information. Facial expressions and intonation was therefore closely observed and scrutinized during conversations. Patients also used the internet to confirm received information and to learn more about the disease

*It was like, straightforward and it’s the kind of doctor that you get an incredible feeling of confidence in right from the start…the most important thing was that it was straightforward so to speak, and not a whole lot of paraphrasing like it maybe was in the olden days (4 M)*

#### Obtaining information as part of a mutual meeting

The patients made a connection between information and the manner in which they were treated by the healthcare professionals. It was very important to be listened to, taken seriously, and to be respected. The quality of the meeting influenced their overall impression of the information they had received. Encounters with the physicians were described as short and rushed by the patients and discharge talks also occurred in the corridor. But some of the patients had a positive experience and were satisfied with the information; this was associated with the consideration that was shown them by that physician.

Experiences of being vulnerable and dependent were also seen in the interviews, and the patients wanted the healthcare professionals to be there for them, to listen and to give information and emotional support.

*You’re so exposed, and you’re like, I don’t know really…you stand alone you feel, you stand alone, and if you don’t get support from those who are supposed to be in the know, well then you feel, you get even smaller…if they don’t listen to you then they don’t…and then you like stand alone once again, it gets even bigger, I mean if you can just talk about it, and just like get it out of your system, then it feels better (5 F)*

### Needing information to manage the worries and make the disease comprehensible

The last category included the following subcategories; *Understanding the cancer disease and what it means, Expressing fear for the future* and *Lacking information leads to anxiety and feelings of insecurity.*

#### Understanding the cancer disease and what it means

When patients left the hospital and returned to their home environments the impact of the disease became very palpable. The focus had turned from being on the coming operation, to absorbing the meaning of the cancer disease and to handling the reactions connected to it. The seriousness of the disease turned their focus towards the end of life. Lacking information on important areas increased the fear.

The information that was generally seen as being the most significant to receive was whether it was a cancer tumour and if any cancer might be left in their body. The patients were ambiguous about receiving information, they wanted to have all information that concerned their own disease but at the same time declared how hard it was to assimilate serious information.

The cause of the disease, how long they had had it, when and where metastases usually turn up and how the disease would be monitored in the near future were expressed as important pieces of information to receive. The results of the surgery and knowing what parts had been removed, was also of great interest.

#### Expressing fear for the future

Patients’ made a connection between the gravity of the disease and the information needed; more serious cancer gave rise to a greater need for information. Receiving information that the cancer had spread to the lymph nodes was described as suffering a setback again, like getting the cancer information all over again. Seeking and receiving information from healthcare professionals was one way of balancing the fear and regaining some control.

*You make a trip through life and there’s a terminal station in life and when you go home with news like this you see the terminal station racing towards you like an express train, so you do everything you can to stand with your hands out to stop the train…so that’s why I want to have information, then I can process it, then I can sort it out into different categories and then I can take care of it (6 M)*

#### Lacking information leads to anxiety and feelings of insecurity

Lacking information about the disease, and the treatment, as well as not having a forecast for the future made the whole situation and the adherent feelings hard to control and manage. It was like living in a state of uncertainty, and the anxiety and the insecurity became even more severe. When a promised appointment did not take place in time, it led to unnecessary anguish for the participant.

*Because that was pure torture for me, to go and wait without knowing…*

*I want to know, because if I don’t know I get insecure and scared and anxious and get all sorts of disturbances, become a pain in the neck for everyone around me…they give you times that they don’t keep, and that, when you’ve got diseases like this one that’s pure psychological terror, I think it’s shocking (6 M)*

Some of them explained that they did not always understand the information they received and this created a similar feeling of insecurity. On the other hand, when healthcare staff could meet the patients’ need for information in time, the anxiety and insecurity became easier to handle.

## Discussion

The patients in this study used information when trying to regain control in their lives after surgery for colorectal cancer. The experience of losing control was related to both the emotional impact of the cancer disease and the bodily changes after surgery, but the lack of, and the waiting for, information enhanced these hardships. To receive and be involved in the information, on the other hand, decreased anxiety and reinforced the feeling of control.

The participants in this study were included by purposive sampling from a larger group of patients who were included consecutively. In this larger group thirty patients declined participation mostly indicating fatigue as the main reason. This could have implications for the results in our study, since those with the worst state of health were not able to participate. These patients’ situation suggests that they are likely to have an even greater need for information and structured discharge planning before leaving the hospital for home.

Carrying out two interviews involved using more of the participant’s time and engagement, and using more resources. This was done to increase the variation in the material by capturing both the initial time just after discharge and the period just after the post-discharge visit at the surgical clinic where the patients received important information about the analysis of the tissue sample [19, 20]. By providing more data in follow-up interviews, the understanding of the phenomenon under study could deepen since the participant would receive more time to reflect upon the questions and could gain more confidence in the interviewer.

One limitation of the study was that some of the interviews were quite short. For example one older participant contributed with a very short interview the second time, answering the questions very promptly. On the other hand, other participants used more time to talk about their experiences and needs of information at discharge, and elaborated on what they had been through during this period. Even though the length of the texts varied between participants the material altogether constituted a rich source for analysis.

Two of three hospitals used ERP and the information was expected to vary somewhat between hospitals. This inclusion was purposeful to increase variation in the material, but the expected disparity was not visible in the text. Unsatisfied information needs after ERP was also seen in another interview study after colorectal cancer surgery [4]. Patients reported a need for more information and easier access to care in the first weeks after discharge.

The use of information to regain control in life after discharge from hospital for colorectal cancer surgery was the main finding in this study. Different areas were identified where patients would need more support in order to regain their control. These were physical, practical and emotional areas, and also the ability to manage the phases and transitions in the cancer trajectory.

The desire to participate in the information was one of the most evident results in this study, and when patients looked back at the discharge and reflected, they could clearly articulate how they wanted to receive information, when to receive it, what it would contain and to whom they wished to talk. These results strengthen other findings suggesting that the key factors for satisfaction are: complete information about the disease and treatment, being treated with respect and empathy, and short periods of waiting [21]. Showing respect and listening actively will enhance communication with patients and their needs will come into focus [2]. This agrees well with patient-centred communication where validating patients’ perspectives and understanding their psychosocial context are necessary to reach the common goal of shared understanding of the patients’ problems as well as shared power and involvement in decisions regarding care and treatment, according to a report from the American National Cancer Institute [22].

Contrary to our results, there are other patients who actually avoid information instead of seeking it in order to cope with a trying situation. If that is mostly due to personality traits or is triggered by the situation is disputed [23]. Most information seekers first use the easiest obtained channel of information without regarding the quality of the source [9]. Accessibility of information is essential not only for seekers of information but also for avoiders that might discover their information needs later on in the cancer trajectory. This increases the demand on healthcare professionals to provide person-centred care with information easily accessible for all patients, even for those who might avoid it at that point. All members of the surgical team need to be involved in the planning and provision of information prior to discharge, but in our findings, the surgical nurses were quite absent in the discharge process. There is room for them to take on a greater responsibility when it comes to the comprehensive structuring of information and discharge planning, and also for involving the patient and next of kin in the process.

The experience of not being prepared to handle the first weeks at home was evident in the results. Different areas that needed improvements were identified, like handling unexpected fatigue and symptoms related to the specific surgery. Being prepared for the next step in the care process was crucial, when the next appointment would be and what the focus of interest would be. Referral to the oncology clinic for chemo-therapy was seen both as a major setback and also as discontinuance in the care process. The need for information is known to differ along the cancer trajectory and if the treatment is finished or on-going [24, 25]. The same was seen in this study where the additional treatment with chemo-therapy clearly increased the need for more information. The findings also emphasized the need to facilitate the transition between clinics. Specialist nurses are known to enhance the transition from hospital to home and between clinics. In a review from the UK the effect of telephone follow-ups for patients with CRC was studied [26]. The results showed that patients received support from a specialist nurse regarding symptom management and emotional reassurance, and they experienced this support as continuity of care. The use of specialist nurses or nurse navigators is widely realized in cancer care [27], but these findings emphasise the need for that role to be more clearly defined and it still needs to be implemented for all patients with different cancer diagnoses and in more countries.

Patients experienced the discharge process as indistinct and it created a feeling of stress, confusion and being left out of the process. A strong need for information and structured discharge planning emerged in the results and the occasional patient who received this declared how much they benefitted from it. These findings are in line with earlier research showing that a discharge planning that is tailored to the individual patient will not only increase participation and satisfaction but will also be more effective when it comes to reducing the length of stay and the number of readmissions [28]. When improving discharge information it is important to consider patients’ context and how this affects their information needs [22]. It is essential to know where in the cancer trajectory patients find themselves at the time, but the physical environment within which the information is communicated is also of importance. To provide a pre-planned appointment in a quiet, separate room that supports communication is a requirement that can be seen as obvious, but quite a few patients are still lacking this context.

After discharge, the main focus changed from the surgery to the cancer itself, and fear and anxiety for the future became more evident. Now was the time to absorb the reality of the cancer disease itself, and it took both time and effort to actually adapt to the word cancer. The anxiety for the cancer and the need to assimilate it in daily life were closely interwoven with getting information about the disease and how serious it was. This is supported by earlier research on communication in cancer care emphasizing the importance of reducing unnecessary anxiety in clarifying what is known about the disease, but also what is not known [22]. In order to help the patient manage the inevitable uncertainty caused by the cancer, an important part of the communication is that of information.

A few individuals in this study could handle some waiting time for information while the majority experienced periods of waiting as unendurable. Waiting time and the need for information is something that surfaces frequently in the literature when patients are asked about what they think needs to be improved in cancer care [21, 29]. Patients are often subjected to waiting for information during the first part of the cancer process, waiting for the diagnosis, the results of surgery, the pathology report and further treatment with chemo-therapy. The findings in our study showed that these periods of waiting were sometimes more in focus than the actual information they were waiting for. The waiting time, without knowing what kind of information to expect next, was filled with worries and many patients described that part as the worst experience in their care process and as being intolerable to endure. These findings suggest that patients need to be more involved and receive more information with regards to what information they can expect to receive and when in the care process they will receive it, in order to increase participation and lessen the negative impact of the waiting times.

## Conclusion

This study showed that receiving information was vital when patients tried to regain control in life after colorectal cancer surgery. The information was necessary in order to facilitate and manage the transition from hospital to home, and the need varied between different transitions. Patients needed more information to manage the daily life at home, but also to understand what the cancer disease really meant to them. This suggests a need for patients to participate more actively in the information and the discharge planning.
